# Cytometric analysis of T cell phenotype using cytokine profiling for improved manufacturing of an EBV‐specific T cell therapy

**DOI:** 10.1111/cei.13640

**Published:** 2021-07-14

**Authors:** Rachel S. Cooper, Aleksandra Kowalczuk, Gwen Wilkie, Mark A. Vickers, Marc L. Turner, John D. M. Campbell, Alasdair R. Fraser

**Affiliations:** ^1^ Tissues, Cells and Advanced Therapeutics Scottish National Blood Transfusion Service Jack Copland Centre Edinburgh UK; ^2^ Blood Transfusion Centre Scottish National Blood Transfusion Service Aberdeen UK

**Keywords:** cell therapy, Epstein–Barr, phenotyping, T cell

## Abstract

Adoptive immunotherapy using Epstein–Barr Virus (EBV)‐specific T cells is a potentially curative treatment for patients with EBV‐related malignancies where other clinical options have proved ineffective. We describe improved good manufacturing practice **(**GMP)‐compliant culture and analysis processes for conventional lymphoblastoid cell line (LCL)‐driven EBV‐specific T cell manufacture, and describe an improved phenotyping approach for analysing T cell products. We optimized the current LCL‐mediated clinical manufacture of EBV‐specific T cells to establish an improved process using xenoprotein‐free GMP‐compliant reagents throughout, and compared resulting products with our previous banked T cell clinical therapy. We assessed effects of changes to LCL:T cell ratio in T cell expansion, and developed a robust flow cytometric marker panel covering T cell memory, activation, differentiation and intracellular cytokine release to characterize T cells more effectively. These data were analysed using a t‐stochastic neighbour embedding (t‐SNE) algorithm. The optimized GMP‐compliant process resulted in reduced cell processing time and improved retention and expansion of central memory T cells. Multi‐parameter flow cytometry determined the optimal protocol for LCL stimulation and expansion of T cells and demonstrated that cytokine profiling using interleukin (IL)‐2, tumour necrosis factor (TNF)‐α and interferon (IFN)‐γ was able to determine the differentiation status of T cells throughout culture and in the final product. We show that fully GMP‐compliant closed‐process culture of LCL‐mediated EBV‐specific T cells is feasible, and profiling of T cells through cytokine expression gives improved characterization of start material, in‐process culture conditions and final product. Visualization of the complex multi‐parameter flow cytometric data can be simplified using t‐SNE analysis.

## Graphical abstract

Adoptive immunotherapy using Epstein‐Barr Virus (EBV)‐specific T cells is a potentially curative treatment for patients with EBV‐related malignancies where other clinical options have proved ineffective. Manufacture of these cells requires robust analytical approaches to ensure quality and efficacy of the cells in therapy. We demonstrate that cytokine profiling can define T cell differentiation status and produce improved characterization of start material, in‐process culture conditions and final product using multi‐parameter flow cytometry and t‐SNE visualisation.

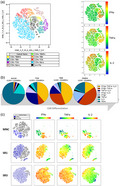

## INTRODUCTION

Epstein–Barr virus (EBV) is a human herpesvirus with a prevalence of more than 90% in adults [1]. Acute infection is generally asymptomatic, but can present clinically as infectious mononucleosis (glandular fever) and infection persists latently throughout life in B lymphocytes. Intermittent viral reactivation drives proliferation of infected cells, which can transform to malignant lymphoblasts in the absence of immune surveillance. In immunocompetent individuals, EBV‐specific T cells control these reactivations [2]. However, iatrogenic immunosuppression of patients following haematopoietic stem cell transplant (HSCT) or solid organ transplant (SOT) can result in potentially fatal EBV‐driven post‐transplant lymphoproliferative disorder (PTLD), where EBV‐transformed B cells develop into an aggressive B cell lymphoma [3].

Adoptive immunotherapy using Epstein–Barr Virus (EBV)‐specific T cell lines has proved to be an effective clinical treatment for patients with rituximab‐resistant or refractory PTLD [4]. The Scottish National Blood Transfusion Service (SNBTS) EBV‐specific T cell bank has delivered third‐party partially human leucocyte antigen (HLA)‐matched EBV‐specific T cells for PTLD patients for more than 15 years, allowing a rapid therapeutic intervention following diagnosis, with an initial 52% complete response rate in a Phase 2 multi‐centre trial [5]. As of December 2020, more than 100 SOT and HSCT patients have been treated with the more recent second‐generation SNBTS T cell bank, issued under a Medicines and Healthcare Regulatory Agency (MHRA) manufacturing Specials licence for therapeutic use. A recent follow‐up study of 64 patients with refractory disease treated between 2011 and 2017 indicates that overall survival at 3 years post‐treatment was more than 40% at 3 years post‐infusion, and even higher (62%) in SOT patients, depending on the degree of HLA matching available [6]. This corresponds well with other clinical studies using adoptive therapy with EBV‐specific T cells, where overall response rates in closely matched donors were 63–68% [7, 8, 9].

Clinical manufacture of allogeneic EBV‐specific T cells for therapy requires a generation protocol that conforms to current good manufacturing process (cGMP) regulations. Manufacture of the current SNBTS second‐generation SNBTS T cell bank was undertaken using a conventional process for generating EBV‐specific T cells, using mononuclear cells (MNC) co‐cultured over 6–8 weeks with autologous EBV‐transformed lymphoblastoid cell lines (LCL). The LCL act as antigen‐presenting cells (APC) to induce proliferation of T cells recognizing EBV viral proteins such as BZLF1 and BMLF1 involved in early lytic cycle transactivation, and viral latency proteins such as Epstein‐Barr nuclear antigen 1 (EBNA1), EBNA2, EBNA3, latent membrane protein 1 (LMP1) and LMP2 [10, 11]. Multiple rounds of stimulation with irradiated autologous LCL are required to induce EBV‐specific T cell expansion to a suitable therapeutic dose [12]. The introduction of rapid expansion protocols in development of new T cell therapies [13] indicates a need for both robust, rapid methods for EBV‐specific T cell manufacture and improved analytical methods to assess the quality of the material throughout.

Replacement of standard flask culture with gas‐permeable rapid expansion devices (G‐Rex flasks; Wilson Wolf, New Brighton, Minnesota, USA) is now established for T cell culture, and T cell lines are generally initiated in culture with a ratio of 40 MNC:1 LCL for the first stimulation round. Subsequent stimulation rounds are generally at a 1 T cell:5 LCL ratio in rapid expansion protocols [14], although variations in this approach are evident between studies [15, 16, 17, 18]. As part of this study, we determined the optimal number of stimulation rounds and optimized our previous manufacture protocol by transfer to European Union (EU) GMP‐compliant culture reagents (medium and cytokine) and G‐Rex flask culture throughout. We confirmed appropriate T cell:LCL stimulation ratio parameters for culture and in each case utilized an extensive multi‐parameter flow cytometric analysis approach to dissect the composition of the cultures.

The critical quality attributes of the SNBTS therapeutic EBV‐specific T cells products are stringent and include viability, T cell lineage and absence of other lymphocytes; for this process optimization study we introduced a broader flow cytometric analysis panel which allowed characterization of the T cell compartment to a much higher level of discrimination. The new panels were able to determine T cell development, differentiation and activation status and provide markers of efficacy through intracellular cytokine analysis for interferon (IFN)‐γ, tumour necrosis factor (TNF)‐α and interleukin (IL)‐2. We utilized these panels to determine the efficacy of the modified protocol in comparison with the current process, and assess quality from starting mononuclear cells through to final T cell product. Visualizing these data is complex, and we applied t‐stochastic neighbour embedding (t‐SNE) algorithms to incorporate multiple parameters into a single image, allowing visual comparison of the T cell phenotype. This approach provides clear evidence for an improved process for manufacture of virus‐specific T cells to a standard suitable for clinical use.

## MATERIALS AND METHODS

### Donor material

Stocks of donor leukapheresis mononuclear cells (MNC)‐derived EBV‐specific T cells and corresponding LCL from the SNBTS second‐generation bank were supplied from frozen stored stocks. For this bank material, initial leukapheresis donations were collected by Spectra Optia apheresis system (Terumo BCT, Lakewood, Colorado, USA) and all donations were fully consented for research use and were either from New Zealand (NZ code, free of variant CJD and suitable for therapy); or were from local Scottish donors for research only. Autologous EBV‐transformed LCL for the second‐generation bank were generated from NZ donor MNC by infection with concentrated EBV‐positive supernatant for use as autologous antigen presenting cells, as previously described [12]. Briefly, supernatant from a live EBV‐expressing cell line was added to donor MNC and cultured for several rounds until stable LCL lines were established. The LCL were frozen in 1 ml aliquots in human serum albumin (HSA) + 10% dimethylsulphoxide (DMSO) until use. Initial work on phenotypical development was performed using MNC derived from buffy coats from normal blood donors, supplied through SNBTS under Sample Governance 14~11.

### Generation of EBV‐specific T cells in standard culture is improved in G‐Rex flasks

Standard cultures were generated by rederiving new products from frozen stored leukapheresis samples from six donors. These new cultures were grown in standard medium [RPMI + 10% fetal calf serum (FCS) and glutamine] in T75 cm^2^ flasks by mixing thawed apheresis MNC with autologous LCL (irradiated at 40 Gy) at an initial ratio of 40 MNC:1 LCL, and then restimulated with fresh autologous LCL for a total of six to eight stimulation rounds. Final T cell products (1.0 × 10^7^ cells/ml) were frozen in cryovials from each stimulation round and stored for later analysis by flow cytometry.

A comparison study set up to assess improvement of culture of EBV‐specific T cells using closable G‐Rex flasks (Wilson‐Wolf) was carried out using thawed donor leukapheresis material and compared against cultures in conventional open culture flasks. Cells in suspension from each donor were split and cultured in either T75 cm^2^ culture flasks (1.0 × 10^8^ MNC + 2.5 × 10^6^ LCL, final density = 1.33 × 10^6^ MNC per cm^2^) or G‐Rex100 cm^2^ flasks (1.0 × 10^8^ MNC + 2.5 × 10^6^ LCL, final density = 1.0 × 10^6^ MNC per cm^2^). At day 10, cells in T75 cm^2^ culture were counted, and a second stimulation of autologous irradiated LCLs were added at a ratio of 4 T cell:1 LCL and split to new T75 cm^2^ flasks to give a final density of 0.5–1.0 × 10^6^ T cells per cm^2^. For G‐Rex cultures at day 10, cells were counted and irradiated LCL added at a ratio of 1 T cell:5 LCL and split to new G‐Rex100 cm^2^ flasks to give a final density of 0.1 × 10^6^ T cells per cm^2^, as indicated in the rapid expansion protocol [14]. At day 14, cultures were re‐fed with IL‐2 at a final concentration of 20 IU/ml. Thereafter, cultures were counted every 3–4 days and fed/split to new flasks as necessary. All cell counts were given as viable cells via trypan blue exclusion of dead cells.

### Comparison of GMP‐compliant reagents and stimulation intensity

Small scale studies of EBV T cells cultured in G‐Rex flasks comparing reagents and stimulation round densities were performed using frozen vials of matched MNC and LCL supplied from the second‐generation SNBTS T cell bank. As before, on day of initiation LCL were irradiated at 40 Gy and mixed with autologous MNC at an initial stimulation ratio of 40 MNC:1 LCL. Cells from each donor were divided and cultured in 40 ml culture medium: either RPMI with glutamine + FCS (10%) + research‐grade IL‐2 (50 IU/ml) or xeno‐free GMP‐grade TexMACS (Miltenyi Biotec, Auburn, California, USA) + GMP‐grade IL‐2 (50 IU/ml) (both Miltenyi Biotec) and transferred to G‐Rex10 cm^2^ flasks (1 × 10^7^ MNC + 2.5 × 10^5^ LCL, final density = 1.0 × 10^6^ MNC per cm^2^). Cell counts were taken every 3–4 days and cultures re‐fed at these time‐points by removing 90% volume of media and replacing with fresh media plus IL‐2. At day 10, cells were harvested from flasks and recultured at 0.5–1 × 10^7^ per G‐Rex10 for restimulation with irradiated LCL at 1T cell:5 LCL, as per standard G‐Rex protocol. Cultures in fully GMP reagents were further subdivided into secondary stimulation ratios of 1 T cell:5 LCL (standard protocol) or 1 T cell:1 LCL (reduced‐intensity modified protocol). A final round of stimulation was performed at day 20, with either 1 T cell:5 LCL or 1 T cell:1 LCL for the standard or modified protocol, respectively. At day 30, cells were harvested from the G‐Rex and assessed for yield and viability by trypan blue exclusion and tested in the flow cytometric assays below.

### Buffy coat MNC isolation

Donor MNC were isolated from healthy donor buffy coats by Ficoll‐Paque (GE Healthcare, Chicago, Illinois, USA) density centrifugation (450 ***g*** for 40 min) to set up flow cytometry panels (surface phenotype and intracellular cytokine assay). Donor MNC from six buffy coats were also characterized for cytokine expression in relation to T cell memory markers (see Intracellular cytokine assay).

### Surface phenotyping

Flow cytometric characterization of T cells was carried out with surface marker panel: CD45RA‐VioBlue, CD8‐VioGreen, CD62L‐fluorescein isothiocyanate (FITC), CD3‐phycoerythrin (PE) or CD57‐PE, CD45RO‐peridinin chlorophyll (PerCP)‐Vio700, CD4‐PE‐Vio770, CD28‐APC (all Miltenyi Biotech) and dead cell marker DRAQ7 (Biolegend, London, UK). Following stimulation by LCL, samples were taken weekly to monitor phenotypical changes using a modified panel, where CD57‐PE (Miltenyi Biotech) was used to replace CD3 to monitor terminal differentiation/senescence.

**FIGURE 1:**
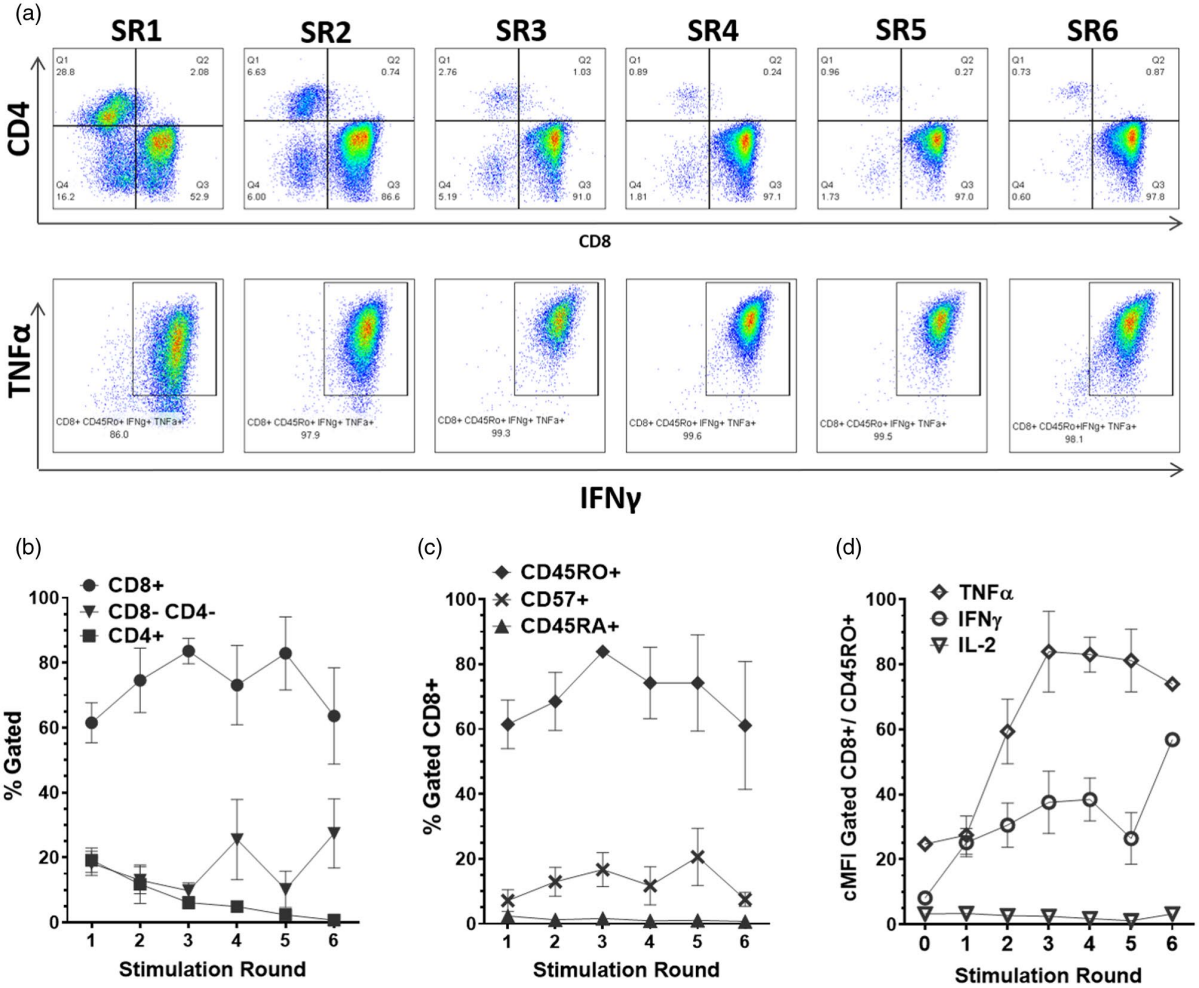
Sequential lymphoblastoid cell line (LCL) stimulation affects Epstein–Barr virus (EBV)‐specific T cell development. Samples were taken 7–9 days after each stimulation round (SR) from six different donor EBV‐specific T cell lines. (a) The T cell ratio shows rapid transformation to a highly enriched CD8^+^ T cell product by stimulation round (SR) 3–4, with most gated CD8^+^CD45RO^+^ cells co‐expressing interferon (IFN)‐γ and tumour necrosis factor (TNF)‐α by stimulation round 2, peaking at SR3. These are maximal cytokine responses driven by phorbol myristate acetate (PMA)/ionomycin. Flow plots are from representative EBV‐specific T cell line NZ873. The mean percentage of (b) T cell subpopulations (CD8^+^, CD4^+^, CD8^−^CD4^−^) and (c) CD45RO^+^, CD45RA^+^ and CD57^+^ in CD8 cells at each SR (*n* = 6) is shown, demonstrating consistent development of CD8^+^ enriched products and consistent differentiation status by SR3. (d) The mean control mean fluorescence intensity (cMFI) of IFN‐γ, TNF‐α and IL‐2 expression in CD8^+^CD45RO^+^ cells reaches a plateau by SR3 then decreases, indicating that peak functionality occurs within three stimulation rounds. Data are represented as mean ± standard error of the mean (SEM)

Briefly, 2 × 10^6^ cells were washed with phosphate‐buffered saline (PBS) plus 2.5 mM ethylenediamine tetraacetic acid (EDTA) (Invitrogen, Carlsbad, California, USA) and 0.5% human serum albumen (has) (Alburex) (PEA buffer), blocked with 5 μl human Fc receptor block (Miltenyi Biotech), labelled with antibody cocktail at 4°C for 20 min, washed and DRAQ7 added prior to analysis. Approximately 100 000 live events were acquired using a MACSQuant10 (Miltenyi Biotech) or BD Fortessa LSR (Becton Dickinson, San Jose, California, USA) flow cytometer with data analysis using FlowJo version 10 software (Tree Star, Inc., Ashland, Oregon, USA). All flow cytometric analyses were based on a manual gating strategy as described in Figure 1a, with lymphocyte population identified by scatter, then doublets and dead cells excluded (singlet gated/DRAQ7^−^ cells). Data were collected as percentages of gated populations as mean fluorescence intensity (MFI), or as a corrected MFI (target MFI–isotype MFI).

### Intracellular cytokine assay

Intracellular staining to characterize T cell populations based on cytokine profile was developed. This approach used phorbol myristate acetate (PMA)/ionomycin stimulation to drive maximal cytokine response in the T cells. Cells were stimulated with 2 μl/ml cell activation cocktail (PMA/ionomycin; Biolegend) for 2 h, then brefeldin A (5 μg/ml, Biolegend) was added for a further 3 h. Non‐activated cells treated with brefeldin A were used as negative controls. Cells were harvested, washed twice in PEA and surface markers labelled with CD8‐VioGreen, CCR7‐FITC, CD57‐PE and CD45RO‐PerCP‐Vio700 (Miltenyi Biotech) as before, washed in PBS plus 2.5 mM EDTA (PE buffer) and stained with 1 μl/ml Fixable Viability Dye eFluor™ 780 (eBioscience, Cheshire, UK) for 30 min at 4°C. Cells were washed again in PE, then fixed and permeabilized as per the manufacturer’s instructions (BD Biosciences) and labelled with IFN‐γ‐eFluor450 (eBioscience), TNF‐α‐PE‐Vio770 and IL‐2‐APC (both Miltenyi Biotech) for 30 min. Cells were washed again and resuspended in PE and up to 100 000 live events recorded on MACSQuant10/BD Fortessa LSR. Further assessment of virus‐specific responses in the T cells was performed in a representative GMP isolate, using stimulation with an EBV‐specific peptide pool (EBV‐Peptivator; Miltenyi Biotech) with PMA/ionomycin as a positive control. The sample was also labelled for markers of cytotoxic function (perforin‐PE and CD107a‐APC).

### Flow cytometry gating strategy and t‐SNE analysis

Phenotypes were defined using a combination of expression of surface markers with intracellular cytokine expression after stimulation with PMA/ionomycin (*versus* relevant negative and isotype controls). Representative flow cytometric data from second‐generation bank EBV‐specific T cell final product with initial gating of lymphocytes/singlets/live cells is outlined in Supporting information, Figure S1. Lineage was identified through CD4 *versus* CD8 labelling and differentiation status through surface expression of CD45RO/CD45RA and CD62L or CCR7 (Supporting information, Figure S1b). Analysis of cytokine expression (Supporting information, Figure S1c) was based on gating CD8^+^/CD45RO^+^ viable lymphocytes, dividing into IL‐2^low^ and IL‐2^high^ populations, and then quadrant gating each IL‐2 subpopulation for IFN‐γ and TNF‐α co‐expression. A contrasting profile of T cell cytokine expression of a heterogeneous pan T cell compartment in MNC freshly isolated from buffy coat donors is shown in Supporting information, Figure S2.

t‐SNE analysis was applied to multi‐parameter flow cytometric data. Briefly, analyses were gated on lymphocyte/singlet/live as above using FlowJo, and reduced to a representative 10 000 events using Downsample (both Becton Dickinson). The t‐SNE maps were generated using the FlowJo plugin for 800 iterations at perplexity 20. Events from each Downsample population were spatially correlated in terms of likeness for all fluorescent parameters. Manual gating overlays were used to subdivide CD8^+^ and CD4^+^ cells into T cell memory populations based on surface marker expression:naive (CCR7^+^CD45RO^−^), T central memory (TCM) (CCR7^+^CD45RO^+^), T effector memory (TEM) (CCR7^−^CD45RO^+^) and terminally differentiated effectors expressing CD45RA TEMRA (CCR7^−^CD45RO^−^).

### Statistical analysis

Statistical analysis was performed using GraphPad Prism version 7.01 software (GraphPad Software, San Diego, California, USA). Comparisons between groups were made on the basis of the difference in mean percentage of cells positive for surface marker expression for phenotypical analyses, the mean fluorescence intensity corrected to negative control (cMFI) of anti‐cytokine antibodies for intracellular cytokine production analyses and the difference in mean cell number/yield for cell expansion analyses. Significance was calculated using unpaired two‐tailed Student’s *t*‐tests with Holm–Sidak correction for multiple tests, where a *p* value of < 0.05 was considered significant.

## RESULTS

### Determining optimal stimulation round for effective EBV‐specific T cell manufacture

Third‐party EBV‐specific T cell banks based on LCL stimulation involve leukapheresis‐derived MNC co‐cultured with irradiated autologous LCL through several rounds to generate a relatively homogeneous virus‐specific T cell product. We hypothesized that multiple rounds of LCL‐mediated antigen presentation could potentially lead to exhaustion of virus‐specific T cells. To determine this, we rederived exemplars from our second‐generation EBV‐specific T cell bank leukapheresis and LCL stocks and analysed cells by flow cytometry at 7–9 days following each successive stimulation round to characterize phenotypical development. Changes in CD4/CD8 ratio were seen through each round of stimulation (Figure 1a), indicating that the CD4 population forms fewer than 1% of the total T cells from stimulation round (SR) four onwards. The corresponding IFN‐γ/TNF‐α expression in the CD8 TCM, TEM and differentiated effector (TEMRA) compartment shows that the majority of cells (86%) co‐express IFN‐γ and TNF‐α in response to PMA/ionomycin stimulation (Figure 1a, lower panel). From SR2 onwards this increases to more than 97% IFN‐γ^+^/TNF‐α^+^, indicating that the CD8 compartment rapidly progresses to functional cytotoxic T cells. However, the percentage of cytokine expression stabilizes after SR3, indicating no significant benefit to functional capacity with multiple rounds of LCL stimulation. This was quantified in six different EBV‐specific T cell lines [data are represented as mean ± standard error of the mean (SEM)], and confirmed that outgrowth of the CD8 population was consistent (Figure 1b). The mean percentage of CD8^+^/CD45RO^+^ cells peaks at SR3 (Figure 1c) then starts to decrease, indicating a trend towards down‐regulation of CD45RO with continued stimulations. Expression of the activation/senescence marker CD57 also shows a marginal increase throughout subsequent stimulation rounds. While the mean percentage of TNF‐α/IFN‐γ‐expressing CD8^+^ T cells does not vary significantly throughout, it is clear that the mean fluorescence intensity corrected to negative control (cMFI) of TNF‐α and IFN‐γ peaks at SR3 or 4 and then starts to decrease (Figure 1d), indicating that extending stimulation beyond four rounds potentially compromises functional cytokine levels. The results from Figure 1 suggest that EBV‐specific T cell cultures reach peak product quality by SR3/4, after which functional parameters decline.

### GMP‐translatable culture vessels improve manufacturing outcomes

**FIGURE 2:**
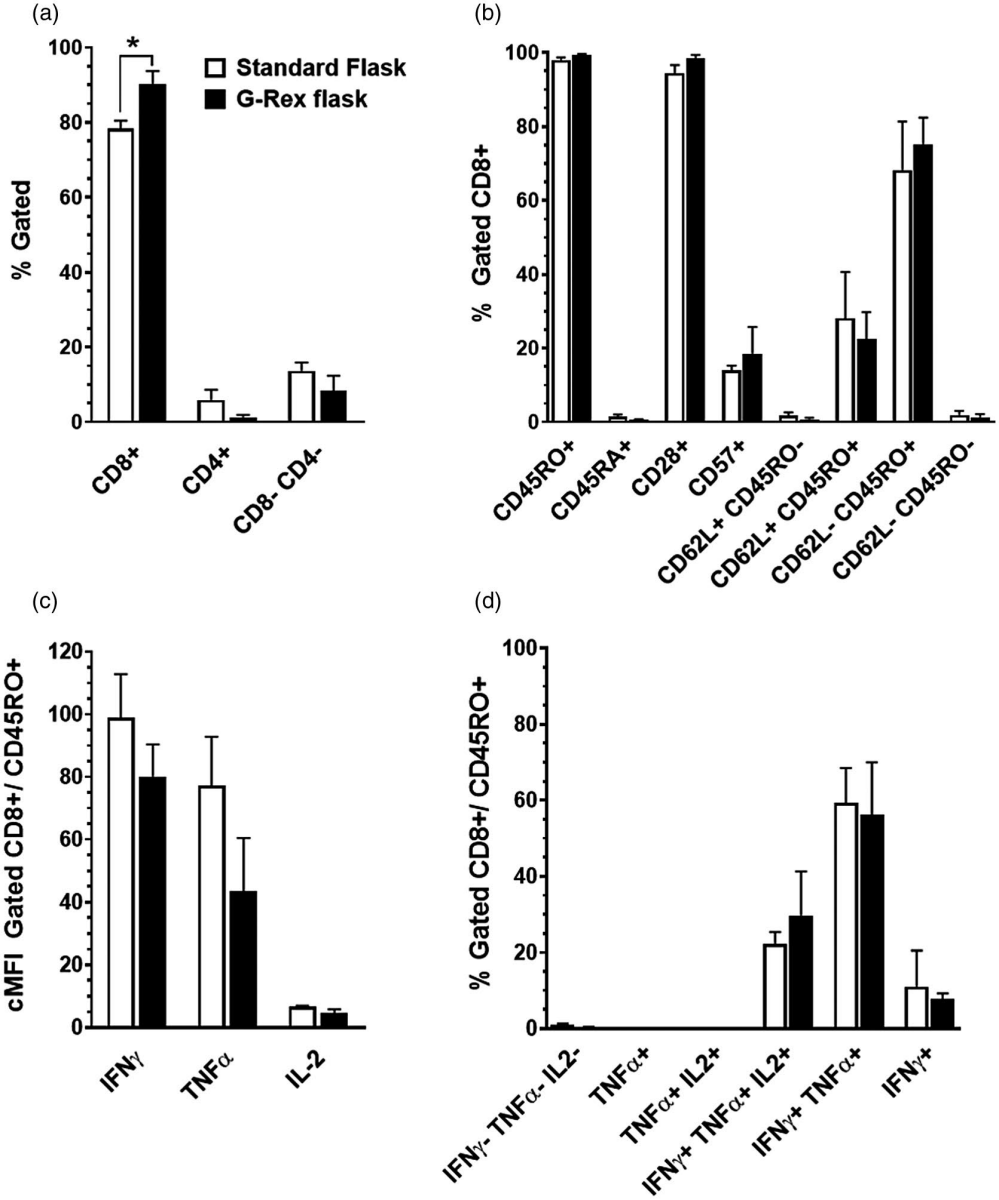
Comparison of culture vessel for T cell products. Co‐cultures of apheresis mononuclear cells (MNC) and autologous lymphoblastoid cell lines (LCLs) were set up (*n* = 6 donors) to compare culture in Corning flasks *versus* G‐Rex100 flasks through an optimized three‐stimulation‐round protocol. Harvested samples were compared for phenotypical and functional profiles, as before. (a) Cultures from G‐Rex flasks had a significantly higher percentage of CD8^+^ cells than standard flask‐grown lines (*p* < 0.05). (b) CD8^+^ cell phenotype was consistent between both culture methods for all markers analysed. Intracellular analysis of the gated CD8^+^CD45RO^+^ cells showed no difference between standard flask and G‐Rex cultured T cells for (c) expression of cytokines by control mean fluorescence intensity (cMFI) or (d) relative frequencies of differentiated subpopulations by cytokine expression. Data are represented as mean ± standard error of the mean (SEM)

The SNBTS second‐generation EBV‐specific T cell bank was manufactured in standard culture flasks, and we determined whether transfer to more GMP‐compliant G‐Rex culture flasks would affect T cell phenotype or functional markers. Apheresis material from healthy EBV seropositive donors (*n* = 6) were used to establish fresh EBV‐specific T cell cultures, and each donor culture was split to directly compare culture in closable G‐Rex flasks (G‐Rex100) *versus* standard tissue culture flasks (Corning, London, UK) through an optimized three‐round stimulation process. The G‐Rex flasks used had a standard research cap, but are identical to GMP‐compliant flasks which have a closed‐process cap, so can be considered GMP‐translatable. At day 30 (9 days after the third LCL stimulation), cells were harvested from both culture vessels and analysed by surface and intracellular cytokine phenotyping. The mean percentage of CD8^+^ T cells was significantly higher (*p* = 0.012) in G‐Rex flasks than in standard flasks (90.2 ± 3.49% *versus* 78.4 ± 2.11%, respectively, Figure 2a). However, T cell surface marker phenotypes did not differ significantly between culture flasks (Figure 2b). Intracellular cytokine analysis after PMA/ionomycin stimulation demonstrated that there was no significant difference in either quantity of cytokine expression (measured by cMFI, Figure 2c) or cytokine co‐expression subpopulations in CD8^+^ T cells between flasks (Figure 2d). These results demonstrate that using manufacturing‐scale G‐Rex culture flasks not only allows increased cell expansion [14], but also develops a more consistent T cell phenotype in a shorter period of time than culture in conventional Corning flasks. This process is then readily transferable to G‐Rex closed‐system flasks to allow a fully closed manufacturing process via use of the GatheRex cell harvester (Wilson Wolf).

### Comparability of EBV‐specific T cells generated using GMP‐compliant reagents

**FIGURE 3:**
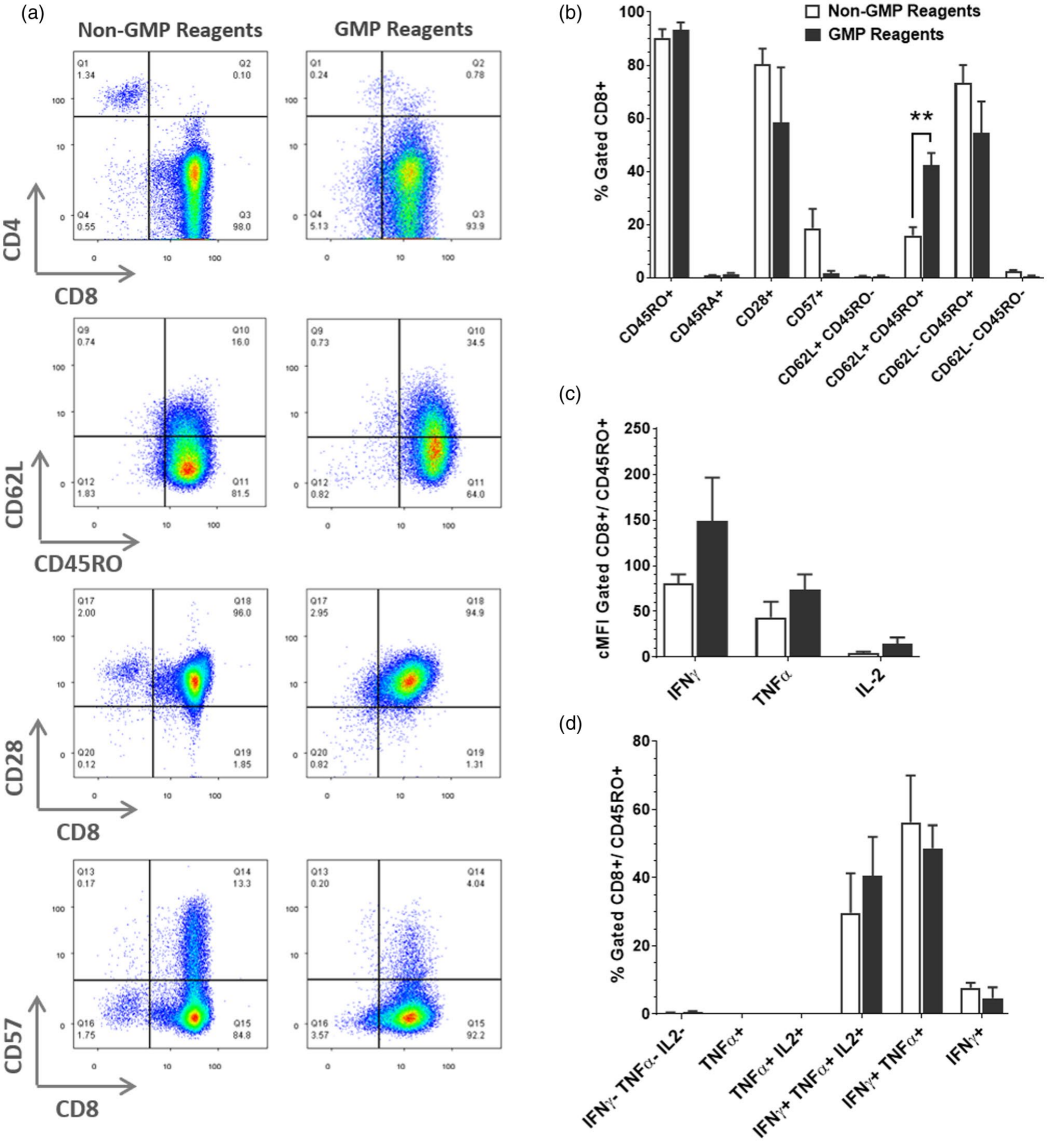
Samples taken at day 30 harvest from G‐Rex10 flasks (stimulation rounds 3 + 9 days) analysed as before. (a) Direct comparison of representative line ladinin 1 (LAD1) cultured using good manufacturing practice (GMP)‐compliant reagents and standard culture reagents demonstrated both T cell cultures had comparable expression of most markers, but reduced CD57 expression in the GMP‐compliant process. (b) Analysis of mean surface marker expression showed a significantly higher percentage of CD62L^+^CD45RO^+^ central memory T cells in GMP‐compliant culture (*n* = 4) compared to standard (*n* = 4, *p* = 0.002). Intracellular staining showed no significant difference in (c) cytokine expression between the two groups or (d) relative frequency of differentiated T cell subpopulations by cytokine expression. Data are represented as mean ± standard error of the mean (SEM)

The SNBTS second‐generation EBV‐specific T cell bank utilized some non‐GMP standard reagents, including FCS for LCL culture. We assessed whether EBV‐specific T cell products cultured in GMP‐compliant reagents including xenoprotein‐ and serum‐free culture medium throughout would generate comparable T cell products. Culture was performed in G‐Rex100 flasks using the optimized three‐round stimulation protocol and assessment of T cell characteristics was carried out as before. In final product, EBV‐specific T cells grown in GMP‐compliant reagents throughout, the surface phenotype (Figure 3a,b) was substantially similar to those grown in research‐grade reagents. A key improvement was a significantly higher percentage of TCM (CD62L^+^/CD45RO^+^) cells in the GMP‐compliant conditions (*n* = 4).

There were no significant differences between the groups for cytokine co‐expressing populations (Figure 3d), although a trend towards increased cytokine levels was observed in GMP‐compliant cultures (Figure 3c). These data demonstrate that GMP‐compliant reagents used with G‐Rex culture flasks results in EBV‐specific T cell cultures that are comparable to current bank material, and have improved retention of the TCM compartment.

### Optimization of T cell: LCL ratio for EBV antigen‐driven stimulation

Conventionally, *ex‐vivo* expansion of EBV‐specific T cells has been performed using a ratio of 40 MNC:1 LCL for the initial stimulation, and subsequent stimulation rounds at a ratio of 1 T cell:5 LCL in G‐Rex rapid expansion protocol, which could potentially drive anergy, senescence or activation‐induced cell death (AICD). We determined whether modulation of the stimulation ratio conferred benefits in terms of functional subpopulation profile.

**FIGURE 4:**
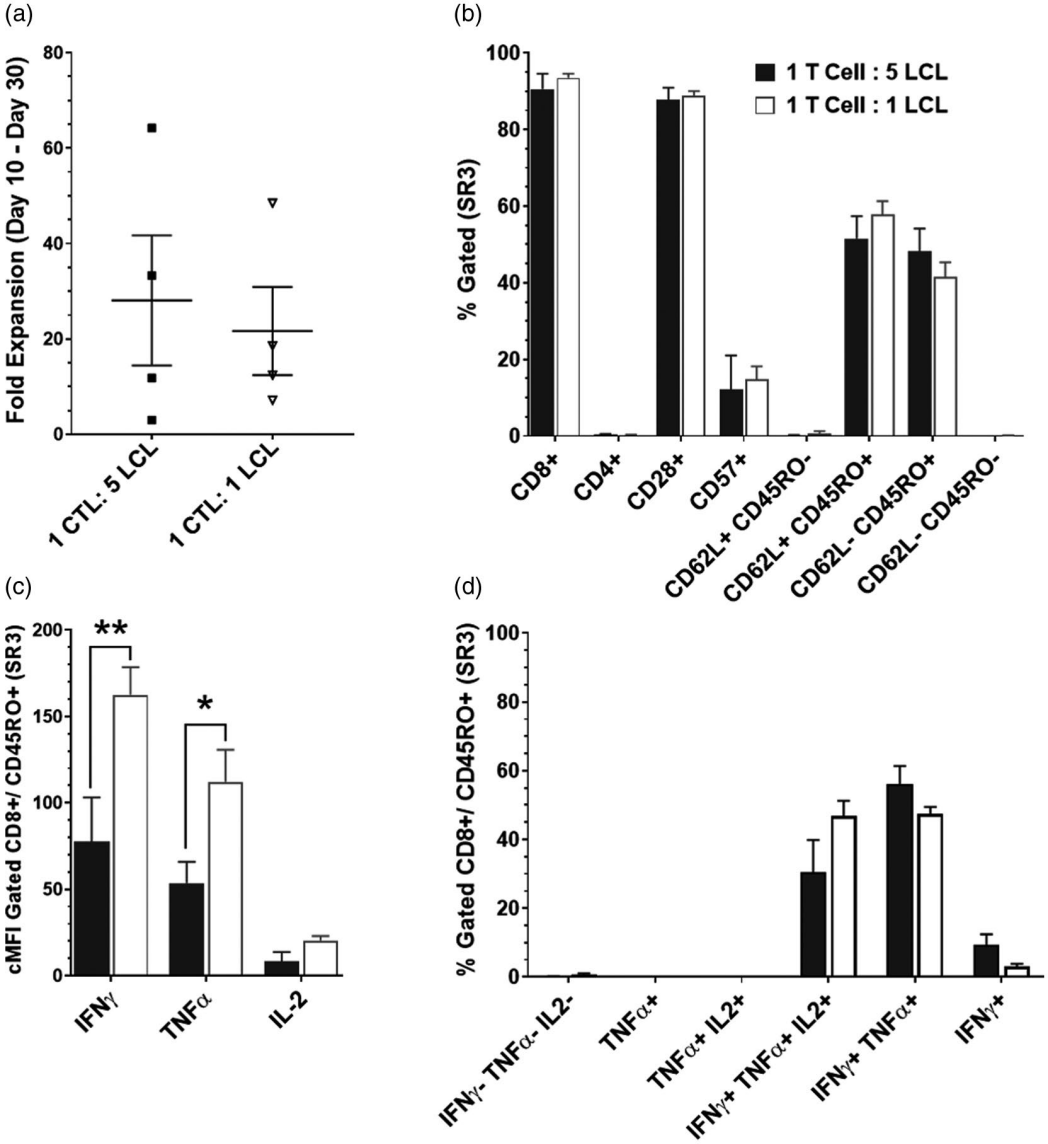
Modifying lymphoblastoid cell line (LCL) [cytotoxic T lymphocyte (CTL):LCL] ratio improves cytokine expression. T cell cultures from four donors were split to directly compare stimulation rounds (SR) 2 and 3 of co‐culture at standard (1 T cell:5 LCL) or reduced intensity (1 T cell:1 LCL). (a) Fold expansion of viable lymphocytes between SR2 initiation (day 10) and end of SR3 (day 30) was calculated from viable cell count. (b) Surface phenotype of final products remain comparable between the two groups for all markers analysed. (c) Reduced intensity culture had a significantly higher control mean fluorescence intensity (cMFI) of interferon (IFN)‐γ (*p* = 0.00218) and tumour necrosis factor (TNF)‐α (*p* = 0.0202) in response to phorbol myristate acetate (PMA)/ionomycin stimulation (corrected to negative controls) compared to the standard ratio cultures. (d) In CD8^+^CD45RO^+^ T cells, there was an even distribution of central memory and effector memory compartments with reduced intensity conditioning, and a skew towards effector memory in standard process. Data are represented as mean ± standard error of the mean (SEM)

T cell cultures from four EBV seropositive donors were initiated using an initial concentration of 40 MNC:1 LCL as per standard protocol, then at SR2 or SR3 cultures were split and co‐cultured at standard (1 T cell:5 LCL) or at lower intensity (1 T cell:1 LCL). At day 30 (the end of SR3), a direct comparison of each stimulation ratio was assessed. Mean harvest cell counts indicated a 28.1 ± 13.63‐fold expansion for 1 T cell:5 LCL cultures and 21.68 ± 9.24‐fold expansion for 1 T cell:1 LCL cultures (Figure 4a). Samples taken at day 30 were comparable for surface marker analysis, with ~90% CD8^+^ cells and equivalent levels of each surface marker subset (Figure 4b). While the percentage of T cells with co‐expression of IFN‐γ/TNF‐α was equivalent between the two groups (Figure 4d), the cMFI of IFN‐γ (Figure 4c) was significantly higher (*p* = 0.00218) in 1 T cell:1 LCL cultures (mean = 162.5 ± 16.03) than in 1 T cell:5 LCL cultures (mean = 77.65 ± 25.50). Similarly, TNF‐α cMFI was significantly increased (*p* = 0.0202) in 1 T cell:1 LCL cultures (mean = 112.18 ± 18.56) than in 1 T cell:5 LCL cultures (mean = 53.69 ± 12.2). The increased cytokine cMFI indicates co‐culture with a lower intensity LCL stimulation result in EBV‐specific T cells with an enhanced capacity for functional cytokine secretion. These data clearly demonstrate the robustness of the current LCL‐based method for EBV‐specific T cell stimulation and expansion, as most parameters are unaffected by the modification of LCL ratios. However, the finding that reducing LCL dose corresponds with increased secretion of IFN‐γ and TNF‐α, which may enhance effector functions of these cells *in vivo*, suggests that the EBV‐specific T cell culture product can be optimized. We have therefore demonstrated that our optimized culture approach provides a significant improvement in retention of TCM cells with increased cytokine expression. In addition, we were able to demonstrate in a representative GMP‐compliant product that there was both clear virus‐specific reactivity in response to a pooled selection of EBV peptides (EBV Peptivator; Miltenyi Biotech) and expression of cytotoxic functional markers (perforin/CD107a) in the final T cell product (Supporting information, Figure S3). The expanded phenotyping approach allows for a better characterization of start material and final products, and we assessed how to analyse the data from this multi‐parameter flow cytometric approach more effectively.

### Comparing T cell memory status using surface marker and cytokine profiles

The flow cytometric characterization of EBV‐specific T cells in the current second‐generation SNBTS bank was restricted to a small panel of essential lineage markers: CD8, CD4, CD19 and CD56 plus 7‐aminoactinomycin D (7‐AAD) for viability. Manufacturing release criteria were confined to the presence of <2% CD19 B cells (as a marker of LCL contamination) and >10% specific lysis against autologous LCLs by cytotoxicity assay. In this study we extended this analysis to characterize T cell products on the basis of lineage, memory and differentiation status, and correlate this with cytokine profile to improve the assessment of quality and potential functionality of T cell material used for clinical therapy. This was used to assess the outcomes from optimization of the LCL‐based manufacturing method.

### Optimized phenotyping and t‐SNE analysis for T cell products

Conventionally, T cell memory subtypes are identified through co‐expression of homing receptors such as CD62L and CCR7 with leucocyte common antigen (CD45) isoforms RO and RA, but these memory types can also be classified by cytokine co‐expression (Supporting information, Figure S4). We characterized T cells from start material through to final product for memory/differentiation status using a combined surface marker/intracellular cytokine panel, and used t‐SNE analysis to simplify multi‐parameter phenotyping.

**FIGURE 5:**
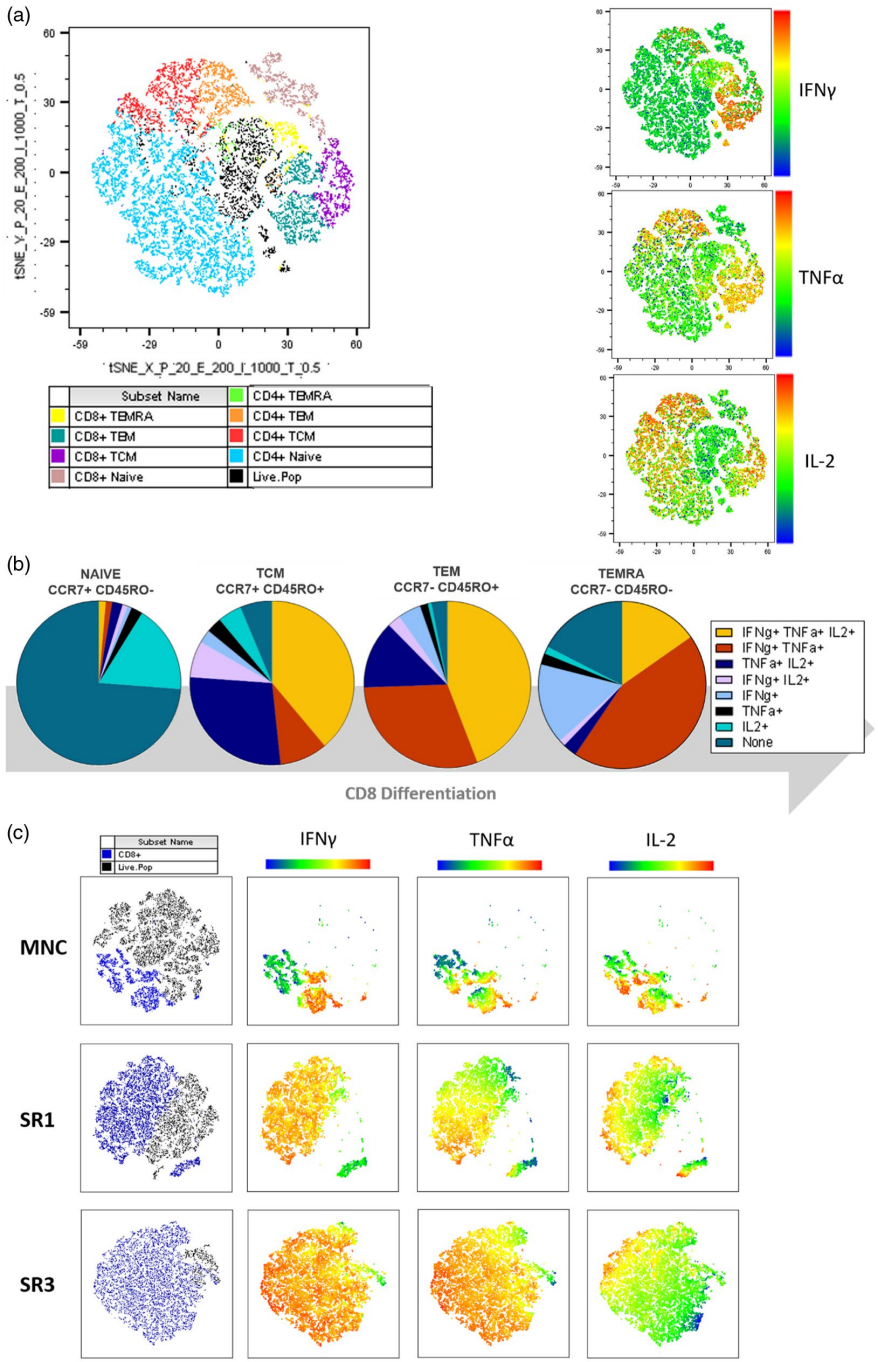
Analytical phenotyping of T cell memory (T_CM_) subpopulations. Differentiation profiles of the T cell compartment were identified by combination of surface marker and intracellular cytokine detection. Donor mononuclear cells (MNCs) freshly isolated from buffy coats were stimulated to induce cytokine expression, and CD8^+^ and CD4^+^ populations were gated into T cell memory subsets dependent upon surface marker expression:naive (CCR7^+^CD45RO^−^), T_CM_ (CCR7^+^CD45RO^+^), T_EM_ (CCR7^−^CD45RO^+^) and terminally differentiated effectors expressing CD45RA (T_EMRA_) (CCR7^−^CD45RO^−^). Stochastic neighbour‐embedding (t‐SNE) analysis was used to cluster cellular subpopulations. (a) Manual gating overlays of an exemplar demonstrate the distribution of CD8 and CD4 memory subtypes in the total lymphocyte population. This same plot was also analysed for individual cytokines by fluorescence intensity. (b) Each subtype was then quantified for expression of single or multiple cytokines, as outlined in the gating strategy. The relative frequency of cytokine subpopulations from representative MNC demonstrates changes in cytokine expression throughout T cell differentiation (mean of *n* = 6). (c) This analysis was applied to our Epstein–Barr virus (EBV)‐specific T cell product using exemplar line NZ873 at optimized culture conditions above [in good manufacturing practice (GMP) reagents within G‐Rex flasks at 1 T cell:1 stimulation round lymphoblastoid cell line (SRL CL) ratio], where there is clear outgrowth of CD8^+^ cells (blue) expressing cytokines from initial MNC starting material throughout the stimulation rounds

The t‐SNE dimensionality reduction identified a clear sequential spatial correlation in both the CD4 and CD8 differentiation from naive (CCR7^+^/CD45RO^−^) to TCM (CCR7^+^/CD45RO^+^) to TEM (CCR7^−^/CD45RO^+^) to TEMRA (CCR7^−^/CD45RO^−^) stages. Furthermore, fluorescent intensity heat maps applied over this same t‐SNE plot (Figure 5b) indicated the highest intensity of IFN‐γ in the CD8^+^ TEM region; the highest intensity of TNF‐α in the CD8^+^ TEM and CD4^+^ TEM regions; and the highest intensity of IL‐2 in the CD8^+^ TCM and CD4^+^ TCM/TEM compartments, confirming the correlation of differentiation status with cytokine profile.

As CD8^+^ T cells form the principal component of the final cell therapy product, the CD8^+^ naive, TCM, TEM and TEMRA populations were then sequentially analysed by expression of cytokines IFN‐γ, TNF‐α and IL‐2. The mean percentage of each cytokine subpopulation from six buffy coat‐derived MNC donors stimulated with PMA/ionomycin is expressed in pie chart format in Figure 5c. The majority of CD8^+^ naive T cells are cytokine‐null, with the remaining 26.2% mainly comprising IL‐2‐ or TNF‐α‐expressing cells. The CD8^+^ TCM cells predominantly express all cytokines (IFN‐γ^+^/TNF‐α^+^/IL‐2^+^), although there is also a distinct TNF‐α^+^/IL‐2^+^ population (27%) within this subset which represents early central memory development. Within the CD8 TEM compartment, there is developmental transition from central memory (retention of IFN‐γ^+^/TNF‐α^+^/IL‐2^+^) into more effector‐like IFN‐γ^+^/TNF‐α^+^ co‐expressing cells, which form almost 50% of the TEM subpopulation. The CD8 TEMRA population shows further transition, with IFN‐γ^+^/TNF‐α^+^‐ or IFN‐γ^+^‐ only T cells forming the principal population, with a small residual population retaining some TCM (IFN‐γ^+^/TNF‐α^+^/IL‐2^+^) characteristics. There is also an increased presence (17%) of cytokine‐null T cells, suggesting that some cells may have become anergic, with no functional secretory response. Importantly, these data demonstrate the limitations of trying to discern T cell memory phenotype purely through surface marker expression alone. For example, even within the conventionally defined naive cells (CCR7^+^/CD45RO^+^), which are typically described as cytokine null, there are evident small subpopulations within that secrete cytokines.

The use of t‐SNE dimensionality reduction generates a single‐image analysis of complex multi‐parameter flow cytometric data which can be utilized to gain a clear representation of the outgrowth of a small discrete T cell population into the final product. Using the data from Figure 1 for sequential stimulation rounds, the t‐SNE plots demonstrate a clear pattern of quality and composition changes from the start material to the final T cell product (Figure 5d).

## DISCUSSION

The SNBTS currently provides allogeneic third‐party EBV‐specific T cells for patients with relapsed/refractory post‐transplant lymphoproliferative disease (PTLD). As of November 2020, more than 100 patients with relapsed or refractory PTLD have been treated from the current bank under a Specials license, with a mean overall survival rate of over 40% at 3 years post‐treatment. Patients in this cohort with PTLD arising after solid organ transplant had better outcomes, with survival of more than 60% at 3 years post‐treatment, and with minimal adoptive cell therapy‐related side effects [5].

The current bank of EBV‐specific T cells was manufactured from 2007 to 2014, and changes to GMP standards since this period have driven a requirement to optimize and refine the current manufacturing processes [19]. More recent methods for generation utilize cytokine release assays to capture virus‐specific T cells, although this requires a different expansion process. LCL‐based stimulation and expansion protocols are still in regular use for development of anti‐cancer therapies [20, 21, 22], and therefore there is a need to identify optimal methods for production and analysis.

In this study we demonstrated that optimization of the standard autologous LCL‐based method of EBV‐specific T cell manufacturing to a fully GMP‐compliant closed‐system process is feasible without compromise in quality of final cell product. The modifications to protocol, reagents and culture process were assessed principally using flow cytometry, which provides a rapid and quantitative method for analysis. Robust, validated flow cytometric assays are a cornerstone of effective reproducible cell therapy manufacture [23]. The use of flow cytometric analysis and functional profiling of EBV‐specific T cells through cytokine expression in this study resulted in improved characterization of both start material and final product and effective assessment of in‐process culture optima, which has been used for analysis of other T cell therapeutics, including a SARS‐CoV‐2‐specific T cell product for COVID‐19 treatment [24]. The improvements in process and final T cell product have been demonstrated within this study, but as this new product has not been subsequently tested in patients we are not able to correlate these improvements with clinical outcome. Further work is in progress to address this issue.

Intracellular cytokine staining for IL‐2, TNF‐α and IFN‐γ provides a reliable method for discriminating the differentiation state of T cells [25, 26]. The combination of multi‐parameter cytokine secretion‐based phenotyping with t‐SNE analysis forms a powerful tool for dissecting subpopulations within the CD8^+^ cytotoxic T cell compartment, and was used as the basis for analysis of improvements and refinements in the manufacturing of the current SNBTS EBV‐specific T cell therapy used for treatment of PTLD [19].

Using the combined surface marker and intracellular cytokine flow cytometric phenotyping approach we were able to identify that multiple rounds of LCL stimulation were unnecessary, and that extending stimulation may increase the level of anergy or loss of function in the T cells, as identified by a loss of absolute IFN‐γ secretion and increased expression of CD57, a marker of terminal effector differentiation [27]. However, the reduction in stimulation round to maximize functional responses needs to be balanced with the requirement for high yields of cells for treatment of multiple patients from a single manufacturing run.

Adoptive T cell therapy relies on large‐scale expansion of functional T cells to manufacture clinically relevant numbers for patient infusion, conventionally through the use of standard culture flasks or gas‐permeable bags. The introduction of large‐volume, high gas exchange culture vessels (G‐Rex flask; Wilson Wolf) has significantly improved the rate and extent of T cell expansion capacity [28]. The G‐Rex flasks are GMP‐compliant and are scalable up to 1‐litre flasks, which are qualified as a Food and Drug Administration (FDA) Class 1 medical device allowing full closed‐process manufacture. This closed‐process manufacture involves suitable sealed flasks, transfer bags, heat‐sealed tubing and the GatheRex cell harvester pump (Wilson Wolf) to ensure sterility in the clinical product. We identified that cell yields could also be improved using G‐Rex flasks for culture with no significant changes in phenotype. A minor change in T cell composition was identified in the G‐Rex cultures, with increase in the percentage of CD8 cells. This consistency of final product phenotype was also retained when all reagents were converted to fully GMP‐compliant standards. GMP‐compliant medium and cytokines with no exogenous xenoproteins ensured that the modified process complied with current regulatory requirements. T cells generated with GMP compliant reagents and flasks suitable for closed‐process culture had a significant increase in retention of the TCM compartment. This has advantages for persistence of the cell therapy once administered to a patient [29, 30].

A principal concern with the current LCL‐based stimulation process is that high LCL (and therefore viral antigen load) ratio to T cells combined with multiple rounds could drive T cell exhaustion [31, 32] and the reduced T cell:LCL ratio process outlined here quantified whether this resulted in functional differences. The LCL process appears robust, as reduced intensity stimulation over three rounds did not significantly affect the phenotype of the T cells at end‐point, although the reduced ratio exhibited a significantly enhanced CD8^+^ cell secretion of IFN‐γ and TNF‐α. The anticipated antigen‐driven exhaustion might be seen at higher LCL to T cell ratios, but further work is required to elucidate this. The only other modulation of culture processes that was not undertaken was to replace or supplement IL‐2 with other gamma‐chain‐specific T cell growth factors. However, other studies have concluded that changing the cytokine‐mediated expansion method from IL‐2 to other cytokines such as IL‐7, IL‐15 or IL‐21 has no significant effect on the overall phenotype or function of T cells for therapy [33]. The increased production of IFN‐γ and TNF‐α in response to stimulation in the reduced‐intensity LCL stimulation may suggest that products made using this protocol could have increased effector functions against viral‐infected cells following patient engraftment.

A key feature of this work was to identify a robust panel of surface and intracellular markers which could effectively classify the T cell differentiation status and development from initial material through to final product. Our approach supplies clear data for this, and demonstrates the utility of this approach for T cell therapies [24]. In addition, the use of t‐SNE dimensionality reduction was very effective at condensing multiple parameters into a single image which could be used to delineate the differentiation stages of the T cells from naive to effector using cytokine expression to enhance standard surface lineage marker data. In addition, the single t‐SNE image approach is a useful graphical representation to identify the status of the material at any stage of manufacture. The images are both illustrative and quantitative and could, therefore, be used as part of a standardized product release process. The data showing the transition of the relevant small T cell component in the starting mixed leucocyte material to its expansion to the final product gives a possible multi‐parameter analysis for quality control as part of a batch manufacturing record. This cytometric phenotype and analysis approach is sufficiently adaptable and inclusive that it would be suitable for phenotypical and functional assay of other cellular therapies, including virus‐specific and genetically modified T cell products [24, 34, 35, 36, 37].

## CONFLICTS OF INTEREST

The authors have no conflicts of interest. This work has not been published previously (except in the form of a poster abstract and MSc thesis), is not under consideration for publication elsewhere and its publication is approved by all authors.

## ETHICS

All donor material was fully consented for research use from New Zealand or Scottish donors, and managed through SNBTS Sample Governance.

## AUTHOR CONTRIBUTIONS

Rachel S. Cooper and Aleksandra Kowalczuk were responsible for the acquisition and analysis of data, and RSC wrote the principal manuscript; Gwen Wilkie and Mark A.Vickers were involved in supply of material and interpretation of data; Marc L. Turner, John D. M. Campbell and Alasdair R. Fraser were responsible for study conception and design, data analysis and interpretation and revision of the final manuscript.

## Supporting information

Fig S1

Fig S2

Fig S3

Fig S4

## Data Availability

Data from this study is available upon reasonable request to the authors.
