# Supplementary figures and images for: Cytometric analysis of T cell phenotype using cytokine profiling for improved manufacturing of an EBV‐specific T cell therapy
Source: Clin Exp Immunol. 2021 Jul 14;206(1):68–81. doi: 10.1111/cei.13640 (PMC8446406; doi:10.1111/cei.13640)

## Slide 1
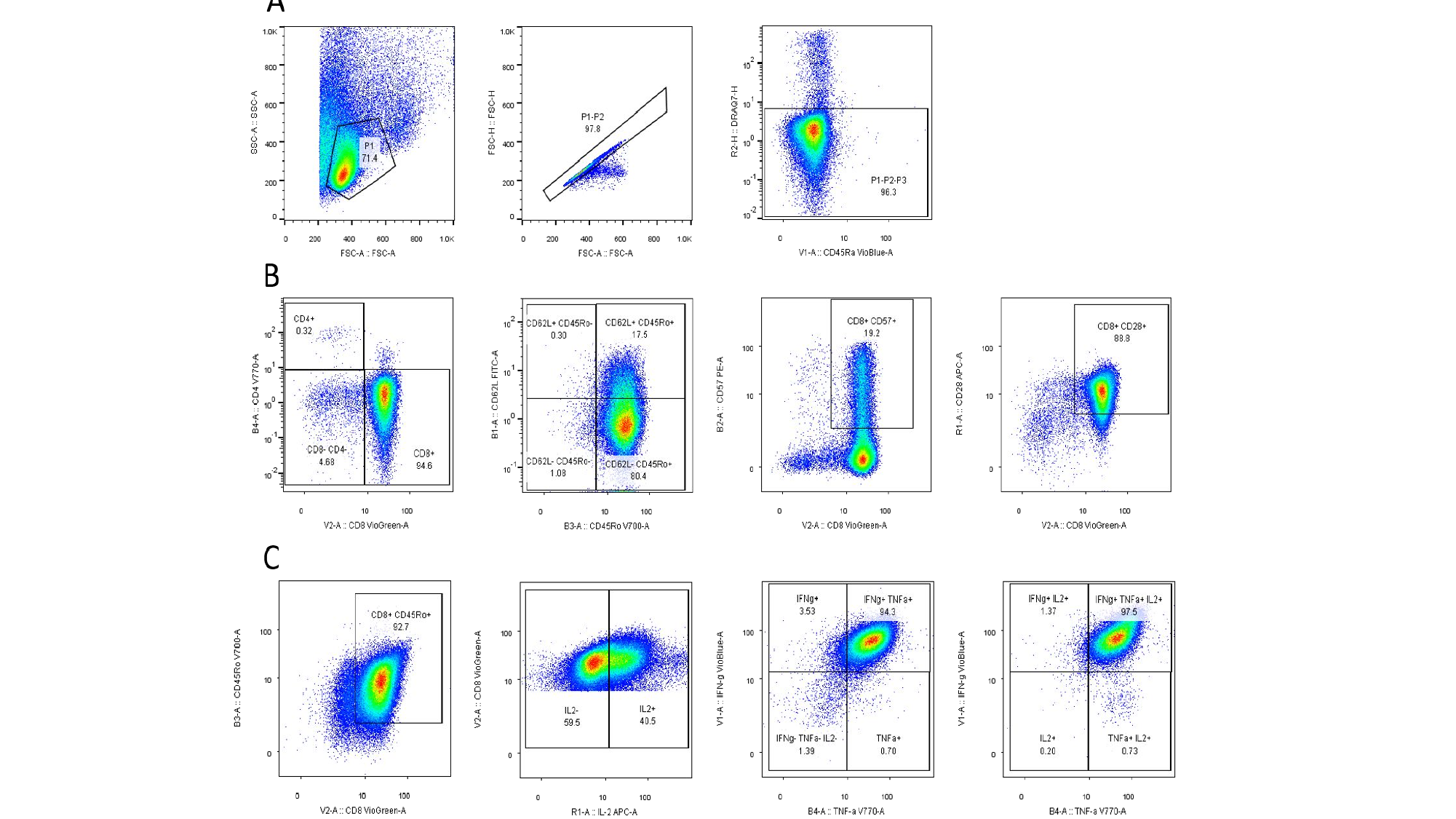

Supplement: Supplementary file 1 — Fig S1 [file CEI-206-68-s001.pptx]

## Slide 1
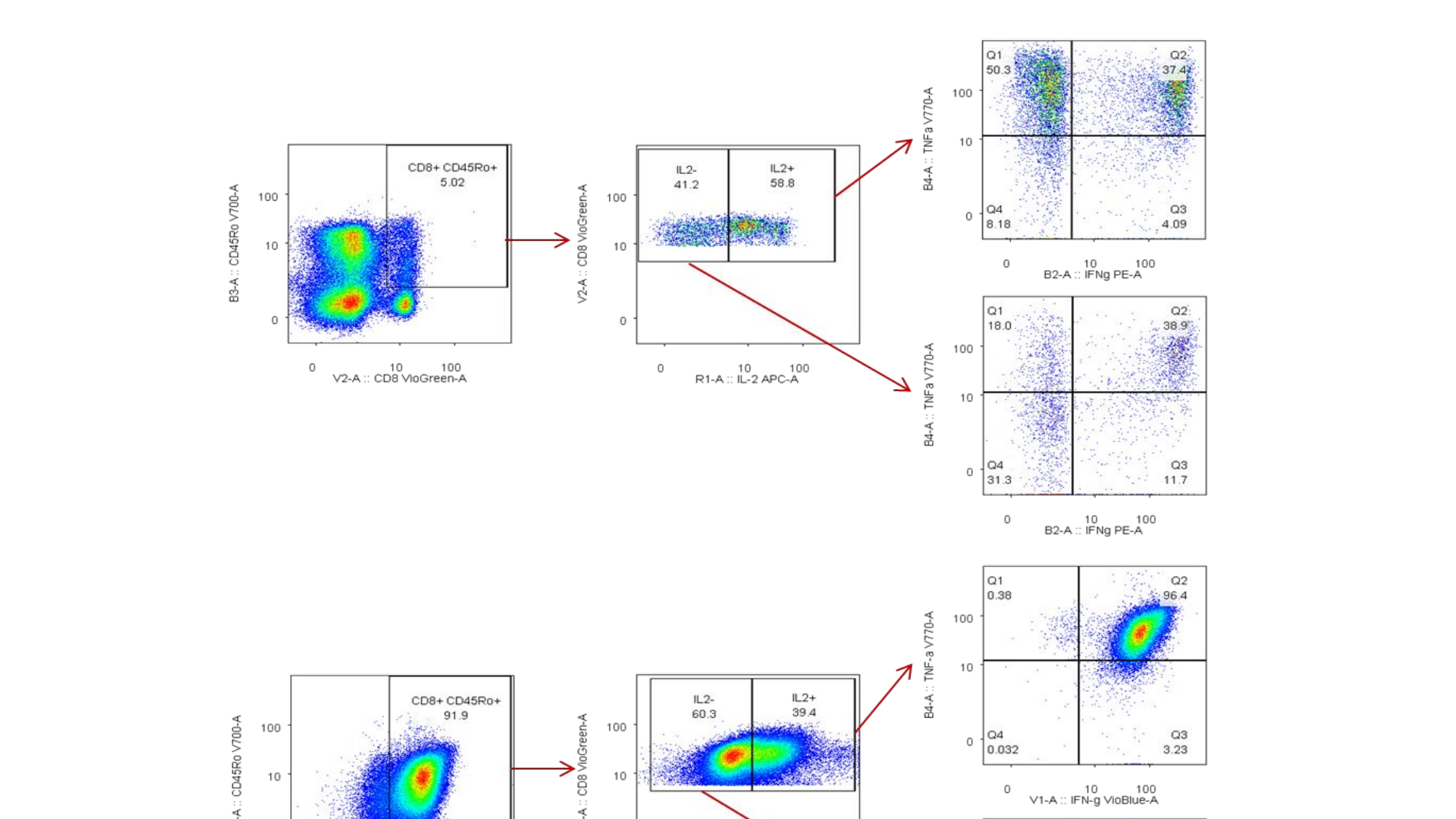

Supplement: Supplementary file 2 — Fig S2 [file CEI-206-68-s003.pptx]

## Slide 1
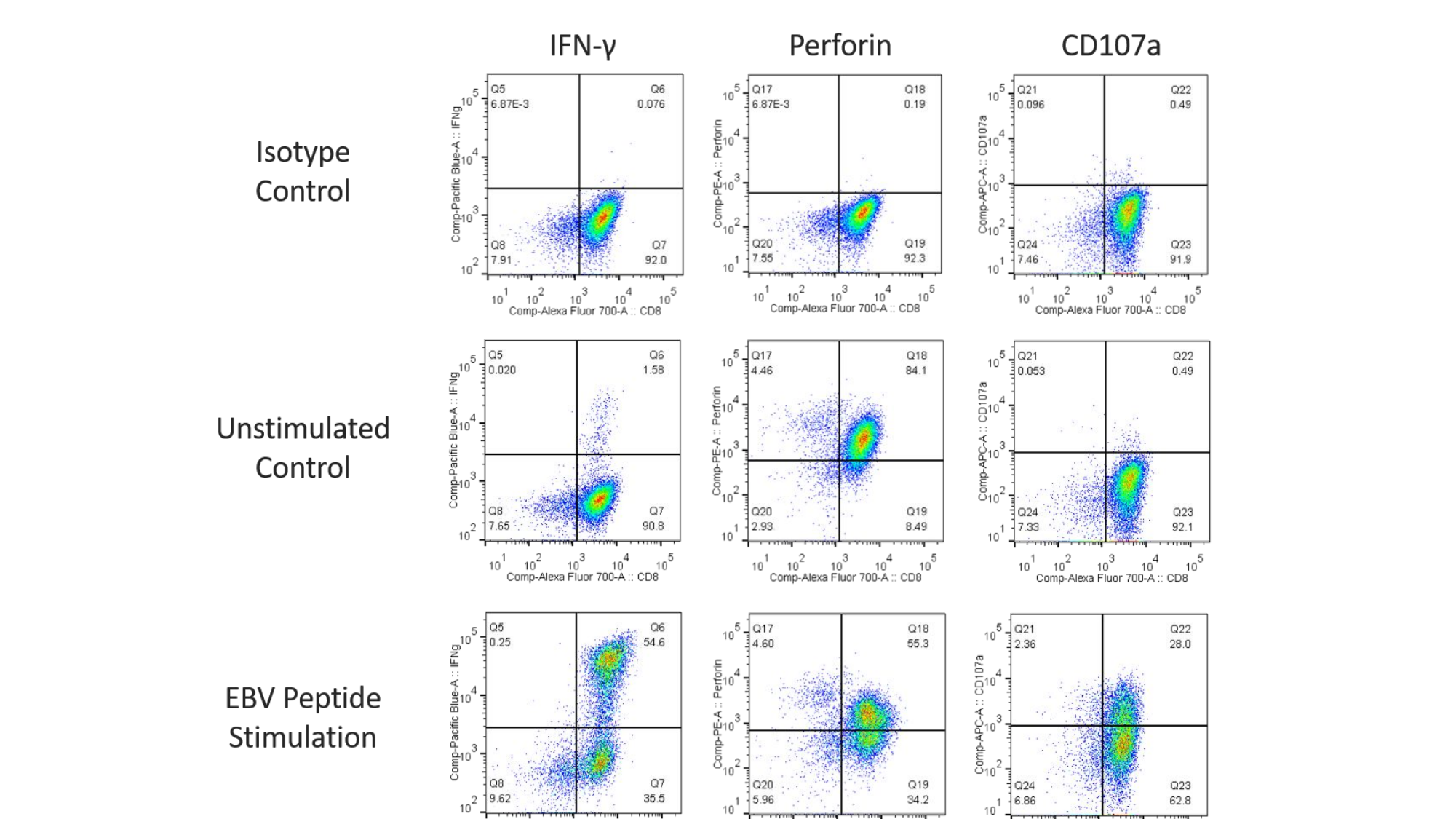

Supplement: Supplementary file 3 — Fig S3 [file CEI-206-68-s004.pptx]

## Slide 1
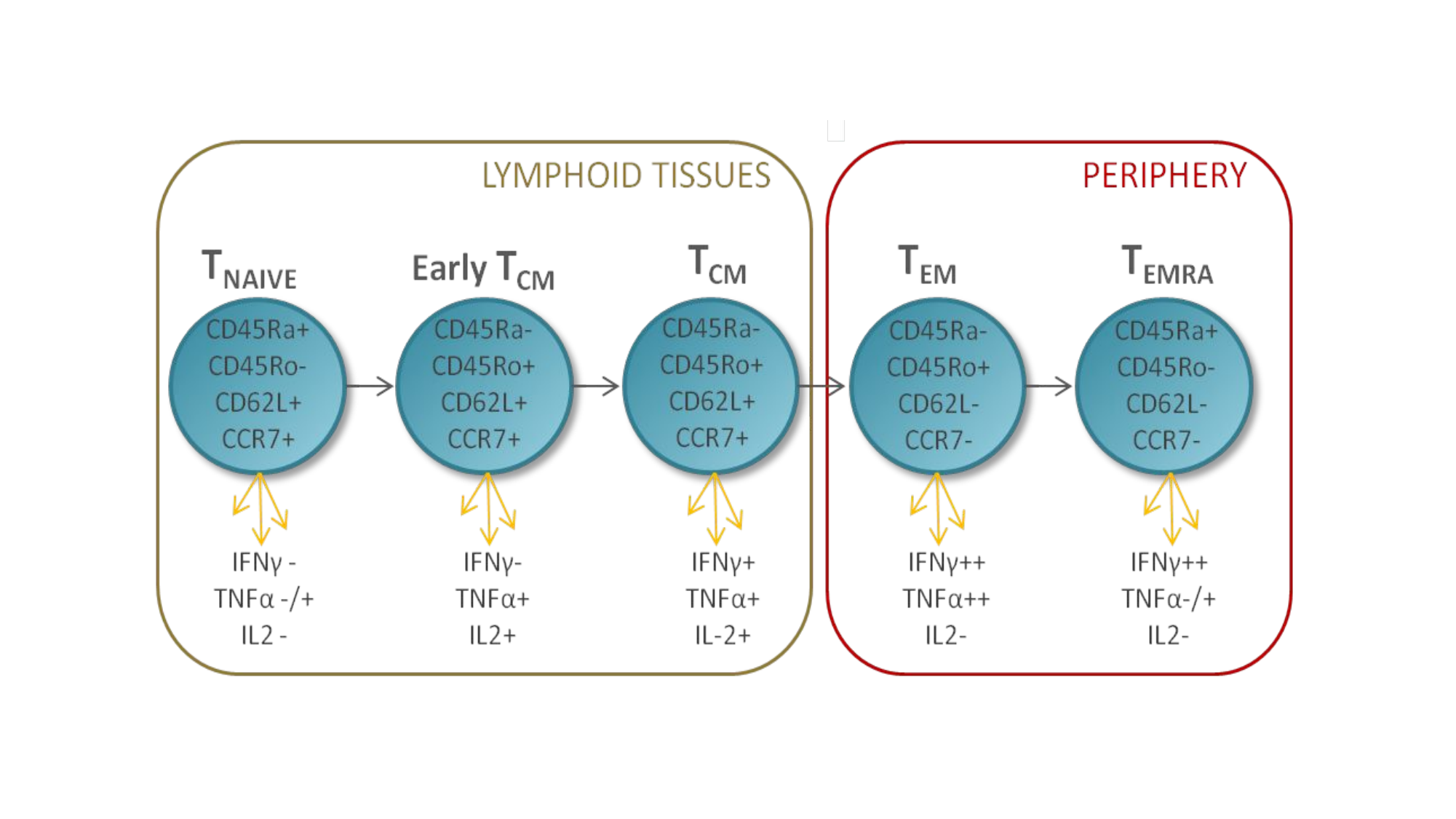

Supplement: Supplementary file 4 — Fig S4 [file CEI-206-68-s002.pptx]
